# Exploring Vibration Transmission Rule of an Artificial Spider Web for Potential Application in Invulnerability of Wireless Sensor Network

**DOI:** 10.1155/2019/5125034

**Published:** 2019-05-19

**Authors:** Jun Wang, Zhuangzhuang Du, Zhitao He, Jiajia Wang

**Affiliations:** ^1^College of Agriculture Equipment Engineering, Henan University of Science and Technology, Luoyang, Henan 471003, China; ^2^Collaborative Innovation Center of Machinery Equipment Advanced Manufacturing of Henan Province, Luoyang, Henan 471003, China

## Abstract

Significant similarities exist between a spider web and wireless sensor network in terms of topology. Combining the unique advantages of the spider web in nature, such as invulnerability and robustness, with communication technology of a wireless sensor network presents high research value and broad development prospects. In this paper, a sort of a spiral artificial spider web based on 3D printing and its associated vibration testing device is proposed, which is used to study the transmission rule of vibration information of the spider web under given excitation conditions. It provides useful inspiration for establishment of an invulnerable communication rule of wireless sensor network. In order to investigate vibration transmission characteristics of the artificial spider web, vibration images are recorded and analyzed by a high-speed photography system, and vibration intensity is characterized by use of peak-to-peak value. Furthermore, vibration performance of the artificial spider web is studied under conditions of integrity and destruction, respectively. Our test observation reveals the vibration transmission rule of the unique structure of the spider web, providing a novel analysis method for improving invulnerability of the wireless sensor network.

## 1. Introduction

A spider web is an essential medium for a spider's hunting and habitation. After billions of years of evolution, it has become an elegant and almost perfect existence. Spider webs can not only maintain strong and effective connections but also quickly perceive vibration information in case of damage to several meshes. The unique mechanical properties and special biological topology of spider webs have attracted attention in this area. Ko and Jovicic studied stress-strain characteristics of the spider web, and the results showed that the spider web features higher toughness than fibers made by current technologies [1]. Yu et al. have successfully simulated the predation process of a spider web by using ANSYS software, analyzing the relationship between the spider web's tension state and energy dissipation [2]. Watanabe discussed whether changing the tension of radial or spiral lines will affect the response speed of spiders, and the result showed that changing tension of spiral lines has little effect on the vibration information perceived by the spider, while increasing tension of radial lines makes it possible for the spider to detect smaller preys [3]. Sadati and Thomas preliminarily investigated morphological computation aspects in naturally spun webs for providing analysis support in the form of a mechanical signal processing system and briefly discussed the web transverse signal filtering, attenuation, delay, memory effect, and deformation modes based on experimental data and numerical simulations [4].

Relevant studies show that network topology of a typical orb spider web is rather similar to the model of a wireless sensor network [5, 6]. To be specific, the spider web can be regarded as a combination of a star network and several ring networks; the hub, as the information center of the spider web, is a typical network with a center; a partially damaged spider web does not affect transmission of vibration signals and prey capture, and failure of partial network components in a wireless sensor network should likewise make no influence on normal operation of the whole network. The transmission rule of vibration information is the external manifestation of unique network topology of the spider web, which can be used as reference for the research of a wireless sensor network with approximate topology. In the past decade, some researchers have made preliminary explorations on the information transmission rule in wireless sensor network inspired by spider webs. Canovas et al. reported an intrusion detection system based on point-to-point communication by imitating the hunting procedure taken by the web spider when it wants to catch its prey, in which the fake wireless sensor nodes were deployed as spiders and the attacker acted as prey [7]. By calculating the physical model of a spider web, Otto et al. established an artificial spider web with diameter being 1.2 m and further studied the frequency response function corresponding to the position of the spider foot under the conditions of different vibration source locations, showing that it is possible to locate the orientation and range of vibration [8]. Liu et al. on the basis of comparing and analyzing the similarity between the spider web and communication network in network elements, communication mechanism, etc., made preliminary analysis on stability of an artificial spider web by use of OPNET software, providing a reference for establishment of a communication-oriented mathematical model of spider web [9]. Wang et al. selected an orb web as the representative of spider web topology types, studied the structure characteristic and invulnerability performance of a spider web, and probed into the possibility of combining robustness and invulnerability of a spider web with farmland wireless sensor network [10]. The existing researches of the spider web-inspired wireless sensor network mainly focus on routing strategy of the spider web, while relatively few of them have thoroughly studied and inherited the communication mechanism of vibration information of the spider web. Meanwhile, several studies have been successfully performed on a self-powered sensor node to overcome energy limitation through piezoelectric material, which highlight the importance of enhancing network communication capability for survivability improvement. Araneo et al. aimed to understand the coupling effects among mechanical, electrical, and piezoelectric properties and presented an accurate numerical modeling of ZnO nanowires, which can provide feasible solution for designing self-powered active sensors to facilitate the wide application range of the wireless sensor network [11, 12].

At present, there are two main potential ways to explore the transmission rule of vibration information of a spider web. One is the software simulation method, namely, the influence analysis of materials with different properties on natural frequency and energy absorption of a spider web by setting up a spider web simulation model. Sensenig et al. showed that energy dissipation of the spider web during prey capture mainly depends on radial lines, while spiral lines and aerodynamic dissipation account for only a very small portion; structure parameters of the spider web directly affect the scope of vibration spreading [13]. Zheng et al. proved that natural frequency and total energy of the spider web can be changed by means of adjusting tension force of radial lines, and the greater the tension force is, the greater the natural frequency and total energy of the spider web [14]. The software simulation method boasts advantages of high speed, flexibility, and low cost, but its simulation results are greatly affected by parameter setting and model construction, so such results cannot comprehensively reflect characteristics of the vibration information transmission of the spider web. The other method is hardware simulation, i.e., simulating the structural characteristics of spider webs in nature by way of artificial web making, using elastic materials such as rubber, resin, and nylon to imitate spider webs, and then studying mechanical characteristics and the vibration information transmission rule of spider web. Eberhard addressed a kind of nylon monofilament-sewn spider web and proposed a new tool for insect sample collection [15]. Qin et al. applied polydimethylsiloxane (PDMS) as the single elastic material to produce spider webs of the same specifications by micro-3D printing technology and studied mechanical response to local loads of spider web with a coarse-grained numerical model [16]. The hardware simulation method obviously provides an efficient and feasible scheme for analyzing the transmission rule of vibration information of spider web.

In this paper, a spiral artificial spider web based on 3D printing is designed, and a device constructed with a high-speed photography system and mechanical auxiliary device for testing vibration of the spider web is used, so as to probe into vibration characteristics of the spider web and summarize the transmission rule of vibration information, providing useful reference for establishing a vulnerable communication rule of wireless sensor network inspired by the spider web. Specifically, under the same excitation conditions, the vibration performance of the complete spider web and spider web with different degrees of destruction is recorded at high speed through marked points, and inspiration value of the vibration transmission rule of spider web to the wireless sensor network is investigated.

## 2. Materials and Methods

### 2.1. Test Device Development

**Figure 1:**
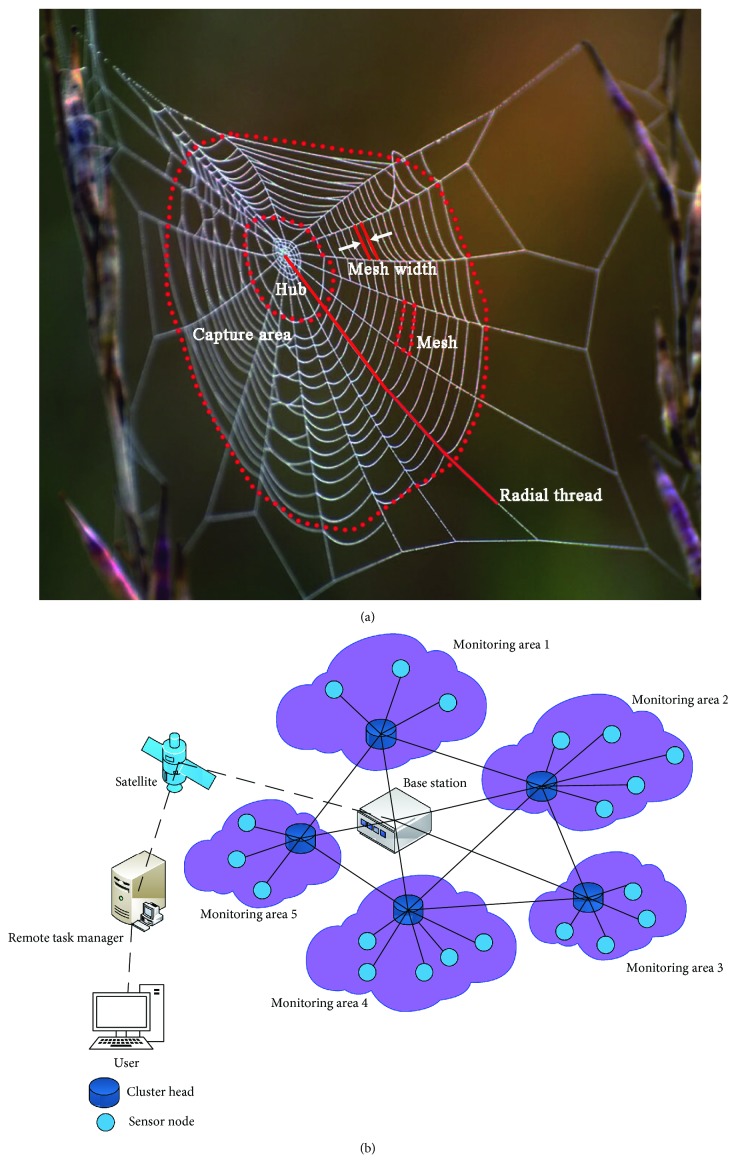
Comparison of the structure between the orb web and wireless sensor network: (a) orb web and (b) layered wireless sensor network system with a center.

The orb web is the most typical representative during evolution of the spider web structure in nature. An orb web is mainly composed of radial threads and capture threads. As shown in Figure 1(a), radial threads radiate outward from the hub, also known as radius threads, which maintain and support stability of the entire spider web structure. At the same time, radial threads feature good ductility and can transmit vibration information in the web [17, 18]. The capture thread is woven out from the hub, with its main role of hunting prey. The architecture form of capture threads reflects the orb web's strategy of prey catching. In general, the diameter and energy dissipation of the capture threads are smaller than those of radial threads [2, 19, 20]. Mesh is the area enclosed by two adjacent radial threads and two adjacent rings of capture threads, and its size represents strength of the spider web. The smaller the mesh, the stronger the spider web. The hub is located at the center of the orb web, where a spider receives the vibration signal of preys. The hub can maintain structure balance of the whole web by equalizing stretching of radial threads. As can be seen from Figure 1, the spiral-layered structure of an orb web is surprisingly similar to that of the layered wireless sensor network with a center in terms of topology and structural function. All the system architecture elements in the wireless sensor network can correspond to certain units or structures in an orb web. For instance, the intersection point of the radial thread and capture thread is somewhat similar to the sensing or relay node in the wireless sensor network; the connection function of the radial thread and capture thread is equivalent to the communication link between nodes; mesh is the same as the block monitoring area surrounded by several nodes; the capture area and the hub act approximately as the whole surveillance area and the base station, respectively. Meanwhile, related research has shown that the spider web has unique advantages in vibration information spreading [21, 22]. Therefore, an artificial spider web vibration test device is developed to clarify and summarize the vibration transmission rule.

**Figure 2:**
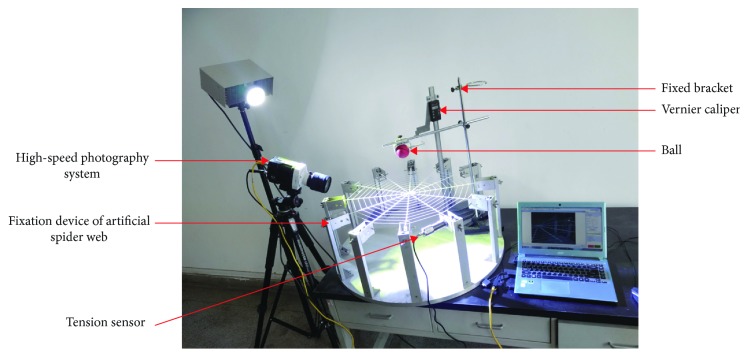
Artificial spider web test device.

The test device consists of a high-speed photography system, fixation device of artificial spider web, artificial spider web, tension sensor, vernier caliper, fixing bracket, and a ball, as shown in Figure 2.

**Figure 3:**
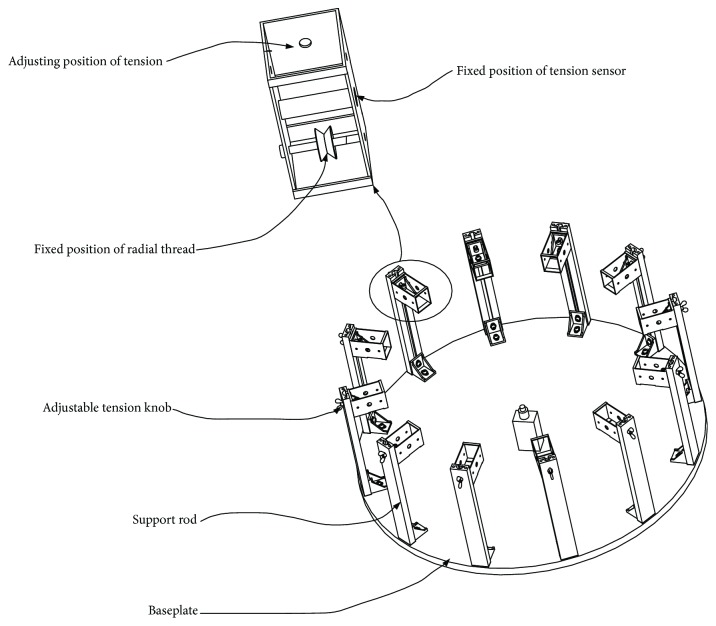
Fixation device of the artificial spider web.

**Figure 4:**
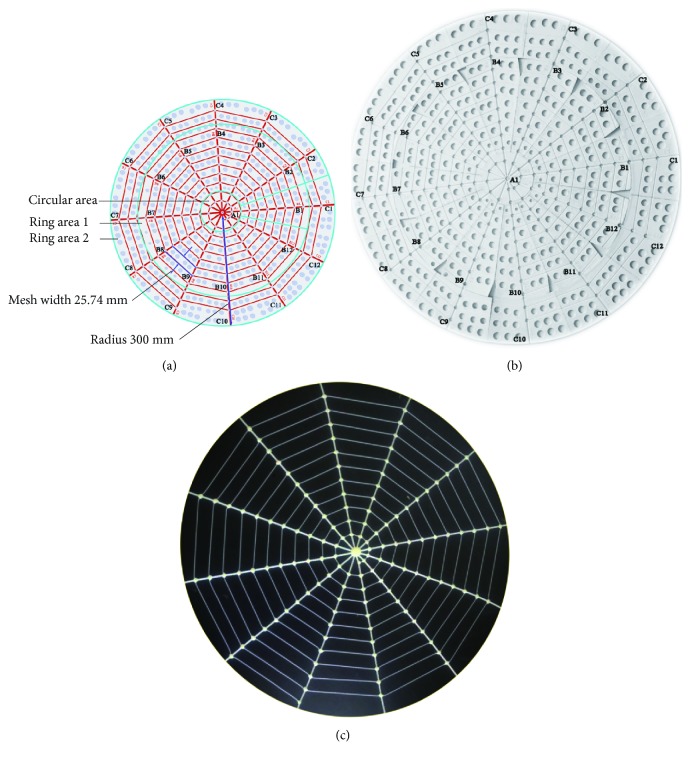
Manufacture procedure of the artificial spider web. (a) Establish the 3D mould of the artificial spider web through SolidWorks software and divide the mould into three kinds of areas; (b) after 3D printing, the artificial spider web mould is assembled; (c) processed artificial spider web.

1. High-speed photography system is composed of a high-speed digital camera (Phantom Miro LC111, Vision Research Inc., USA) and digital image acquisition system. The minimum exposure time of the high-speed digital camera is 2 *μ*s, the resolution set for the test is 1024^∗^768, and the shooting speed is 100 frames/s. A laptop computer (PC-20160811UXVU, Acer Inc., China) is used to record data with a processor of Intel Core i3-2367M and a RAM of 4 GB
2. As shown in Figure 3, the fixation device of artificial spider web consists of a circular aluminum baseplate with a diameter of 800 mm and a thickness of 20 mm and 12 aluminum support rods of the same specifications. The height of these rods is adjustable. Moreover, the design includes fixed position of the radial thread and single pulley tension sensor and the adjustable tension knob. The tensioning force of radial threads can be measured and adjusted. The tension of the radial thread is 2.5 ± 0.2 N
3. The artificial spider web is composed of 12 radial lines (radial threads) and 11 layers of spiral lines (capture threads). There is a circular groove with a diameter of 3 mm at the position of the radial line and a groove with a diameter of 1.5 mm at the position of the spiral line. As shown in Figure 4, manufacture procedure of the artificial spider web is divided into 3 steps: (1) Design the 3D mould of the artificial spider web. The mould features an equidistant spiral line amplification structure, with spacing being 25.74 mm; the whole web's radius is 300 mm, and its area is 282,600 mm^2^. After coding the mould, divide it as follows: The circular area includes module A1; ring area 1 includes sector modules B1-B12, and ring area 2 includes trapezoid modules C1-C12. (2) Use a 3D printer (Z603Sn Jgaurora Ltd., China) to print the artificial spider web mould with photosensitive resin material. After printing, assemble the complete artificial spider web mould according to numbering. (3) Apply round strips of silica gel with diameter being 3 mm and tensile strength 20 N/cm and those with diameter being 1.5 mm and tensile strength 10 N/cm to be extruded into the grooves, respectively, and then, apply 706 silicone rubber to intersecting position of the two kinds of silicone strips to glue the web lines together. Firstly, a small amount is evenly applied to fixation joints; after 5-6 hours for full solidification, the spider web is suspended for the second gluing to increase tensile strength. Generally, tensile strength can reach 15 N/cm
4. Single pulley tension sensor (Bangbu Sensor System Engineering Ltd., China) is selected with a measuring range of 0-10 N and an accuracy of 0.01 N. The height measurement adopts a vernier caliper with digital display (Guilin Guiliang Tools Ltd., China), whose range is 0-500 mm, and its accuracy is 0.01 mm. A contraction clamp is used to fix and release the ball, and an adjustable knob is used to fix the contraction clamp onto the vertical bracket. The height adjustment range of the bracket is 0-1000 mm for tuning and fixing falling position of the ball. The ball is a rubber ball with a mass of 20 g and a diameter of 50 mm

### 2.2. Coding Rules

**Figure 5:**
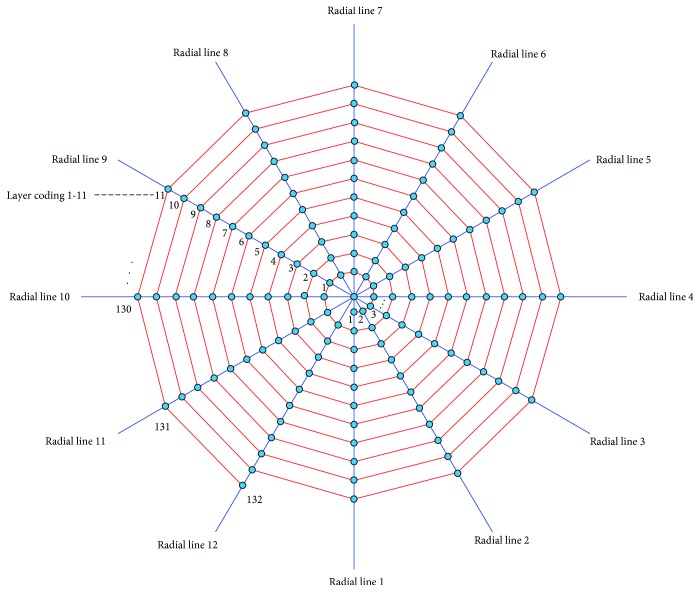
Coding rules of the artificial spider web model.

The vibration test of the artificial spider web has to track and analyze vibration of nodes, radial lines, and spiral lines. We establish a set of sound coding rules to differentiate them.  Figure 5 demonstrates the artificial spider web coding rules, in which radial lines and spiral lines are represented as radial lines 1-12 and spiral lines 1-11, respectively, with radial lines as horizontal ordinates and spiral lines as vertical ordinates. The nodes where radial lines intersect with spiral lines are coded by way of the coordinate point marking method in a plane coordinate system. In the direction of spiral amplification, the nodes are numbered 1-132 in sequence from inward to outward. So for a node coded *n*, its horizontal ordinate is the remainder obtained by comparing *n* and the total radial line number, while its vertical ordinate is 1 plus the integer of the remainder obtained by comparing *n* and the total radial line number. This numbering method can accurately locate any node on the artificial spider web. According to the definition, starting with node 1, along the direction of spiral amplification and for each 12 nodes, the coded number of spiral lines increases by 1, so there are 11 layers in all; the radial line passing node 1 is defined as radial line 1, and the coded number of radial lines in a counterclockwise direction increases in turn, with a total of 12 lines.

### 2.3. Analysis Method

In the trial, the ball is released freely from different positions 30 cm above the artificial spider web surface; when it bounces up, it is manually caught to make sure that the ball will not fall back to the artificial spider web. The influence of different types of spider web structural components and different damage patterns on vibration transmission is, respectively, studied. In order to improve test accuracy, all the tests were repeated three times. The recorded vibration images of the artificial spider web are analyzed by high-speed photography analysis software PCC 2.6, thus tracking the motion of each marked point and obtaining vibration data continuously. The vibration data are processed by Excel 2010, and vibration of marked points is analyzed by use of peak-to-peak value. Significance and correlation of vibration data are analyzed by SPSS Statistics 24.0.

## 3. Result and Discussion

### 3.1. Complete Web Test

**Figure 6:**
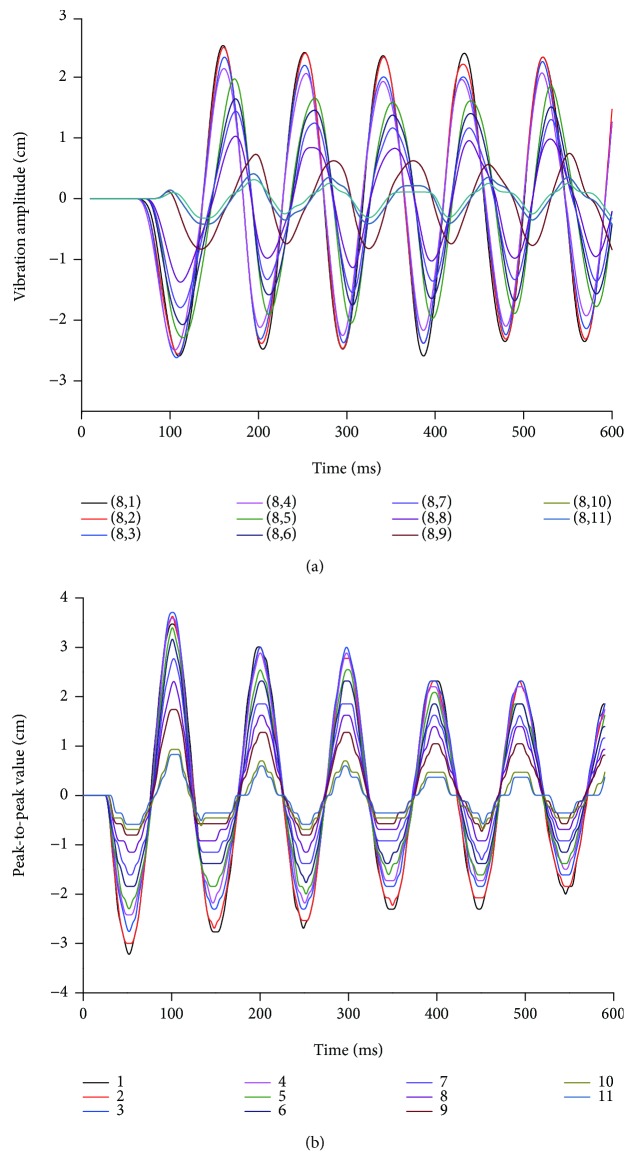
Waveform of node and spiral line vibration: (a) amplitude variation curve of longitudinal vibration of nodes on the 8th radial line; (b) amplitude variation curve of longitudinal vibration of layers 1-11 of spiral lines between 8th and 9th radial lines.

When the ball falls freely 30 cm above the center of the artificial spider web, the longitudinal vibration waveform (perpendicular to the plane of the artificial spider web) is formed by the impact to the web according to Figure 6. It can be observed that there are always delays in oscillation startups of nodes and spiral lines from the inner layer to the outer layer, and the delay of node oscillation starting is obviously greater than that of spiral lines; on the same layer, vibration intensity of nodes is greater than that of spiral lines; hence, nodes transmit more vibration energy than spiral lines. As shown in Figure 6(a), the vibration gradually attenuates from the inside out. The peak-to-peak value of vibration near the center is about 8 cm, and the energy attenuation increases with the distance from the center. When the vibration reaches nodes of spiral lines on 10th and 11th layers, peak-to-peak value of the vibration is about 1.5 cm, only 1/6-1/5 of the peak-to-peak value of the center. The comparison between Figures 6(a) and 6(b) shows that vibration attenuation speed of spiral lines is obviously faster than that of nodes. By the 6th cycle, peak-to-peak value of the spiral line vibration at the same position has attenuated by about 1/3, while the change of nodes on the radial line is not significant. The artificial spider web features the longitudinal vibration delay rule and rule of attenuation. These rules are similar to network delay (channel access delay, transmission delay, and transmitting/receiving component startup delay) and information transmission signal attenuation in the wireless sensor network [23–25]. In addition, the difference between vibration intensity of nodes and the spiral lines shows that the radial line where the node is located is the main path for vibration transmission while the spiral line is the auxiliary path. This is similar to the configuration of primary routing and auxiliary routing in data forwarding of the wireless sensor network.

**Figure 7:**
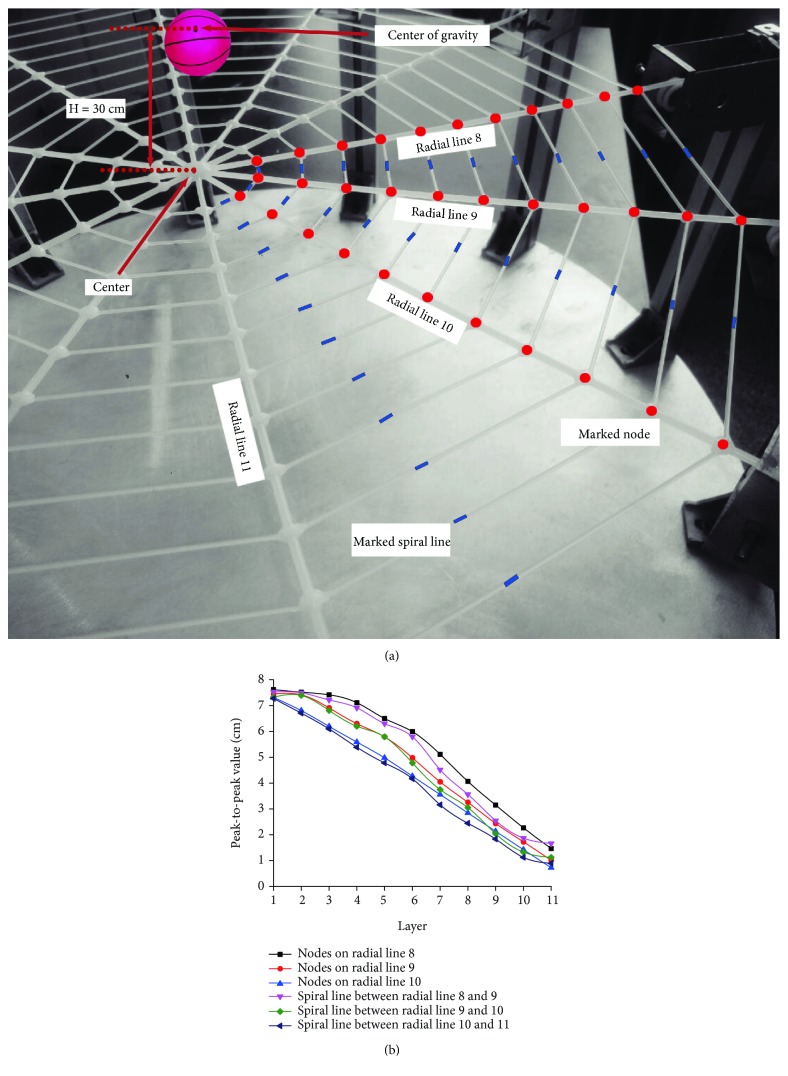
Variation of longitudinal vibration of 33 nodes on radial lines 8-10 and spiral lines between radial lines 8 and 11 in response to the central vibration source: (a) distribution of nodes and spiral lines tracked; (b) curve of peak-to-peak values of longitudinal vibration of nodes and spiral lines tracked in response to change in the layer number.

When the ball falls freely 30 cm above the center of the artificial spider web, the peak-to-peak value of longitudinal vibration of the 33 nodes on radial lines 8-10 and spiral lines between radial lines 8 and 11 is shown in Figure 7. This shows that with the increasing of the layers, vibration intensity of nodes and spiral lines decreases gradually; the peak-to-peak value of the nodes on radial lines 8-10 attenuates from about 7-7.5 cm of the first layer to about 1-2 cm of the 11th layer; amplitude attenuation speed of inner nodes on radial lines 8-10 is obviously lower than that of outer nodes. The peak-to-peak value of the spiral line decreases from about 7.3 cm on the first layer to about 1 cm on the 11th layer, slightly smaller than the vibration peak-to-peak value of the node on the same layer. It is found that longitudinal vibration sustained by nodes and spiral lines on inner layers of the artificial spider web is, respectively, much greater than that by those on outer layers; the layer position and vibration transfer function of the node on the inner-layer hub are similar to that of the cluster head; in the layered structure of the wireless sensor network, the cluster head is closer to the base station geographically, and the amount of information it receives and transmits is much greater than that of the peripheral common nodes [26]. The vibration forwarding function of inner spiral lines is equivalent to the peer-to-peer efficient communication link between cluster heads, while the outer spiral lines are similar to the auxiliary communication link among the common nodes for ensuring network connectivity and reducing communication congestion.

**Figure 8:**
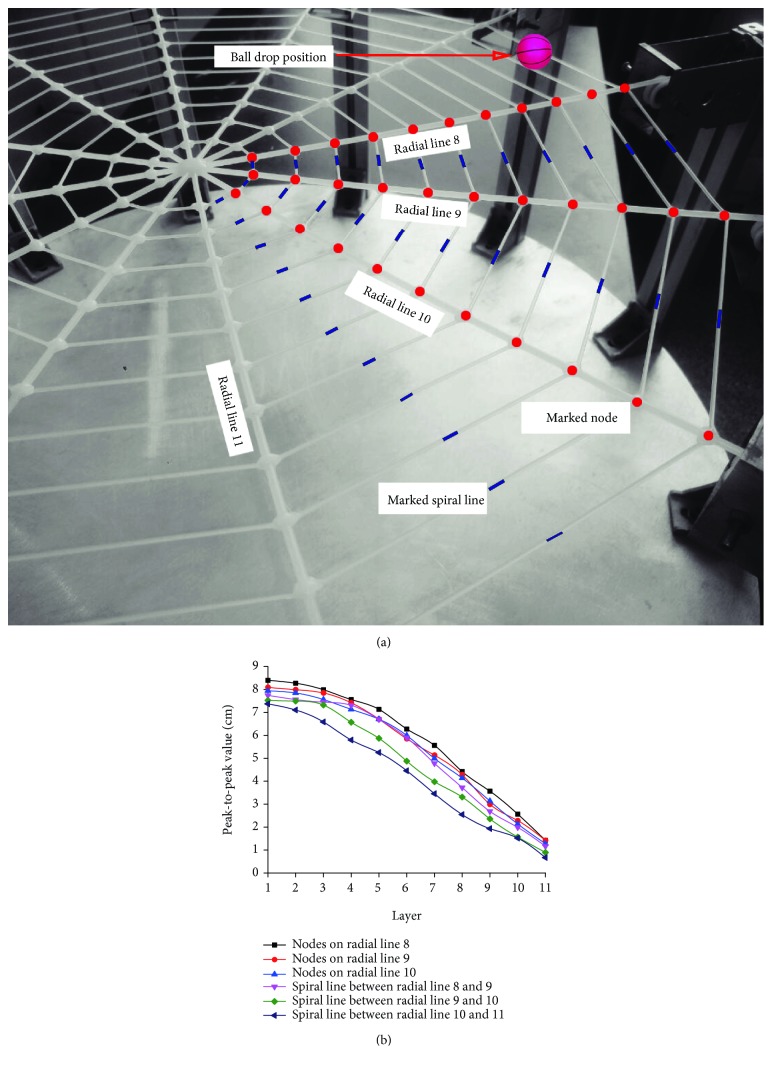
Variation of longitudinal vibration of 33 nodes on radial lines 8-10 and spiral lines between radial lines 8 and 11 in response to the edge vibration source: (a) distribution of tracked nodes and spiral lines; (b) curve of peak-to-peak values of longitudinal vibration of tracked nodes and spiral lines in response to change in the layer number.

Figure 8 shows when the ball falls freely 30 cm vertically above the meshwork surrounded by nodes (7,10), (7,11), (8,10), and (8,11) and the peak-to-peak value of longitudinal vibration of the 33 nodes on radial lines 8-10 and spiral lines between radial lines 8 and 11. It can be found that the vibration rule of nodes and spiral lines is basically consistent with the rule when the ball is released at the center point; in both cases, vibration intensity of outer layers is less than that of inner layers. The test result shows that the unique structure of the spider web has a central agglomeration effect on vibration. This is similar to node deployment and the network information transmission mode based on PEGASIS and LEACH_EE protocols in the layered wireless sensor network with a center [27, 28], namely, the information collected by outer common nodes is first transferred to cluster heads, and cluster heads forward data to the base station. The amount of information received and transmitted by the inner network is much greater than that by outer common nodes, and deployment density of inner nodes is higher than that of outer nodes.

**Figure 9:**
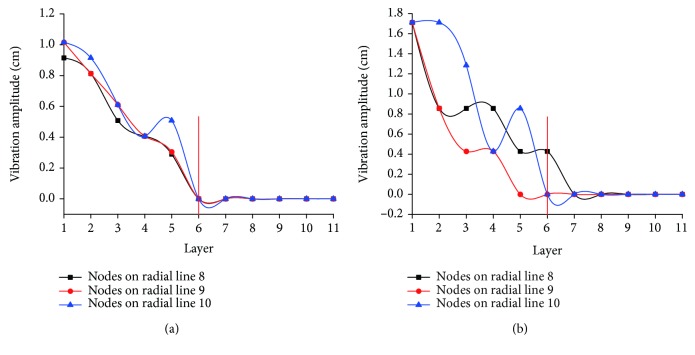
Variation of horizontal vibration of 33 nodes on radial lines 8-10 in response to vibration sources on varied positions. (a) Peak-to-peak variation curve of horizontal vibration of nodes in response to the central vibration source; (b) peak-to-peak variation curve of horizontal vibration of nodes in response to the edge vibration source.

Figure 9 shows when the ball is, respectively, released above the center and outer mesh locations and the peak-to-peak value of horizontal vibration (parallel to the plane of the artificial spider web) of the 33 nodes on radial lines 8-10 being tracked. It can be seen that the artificial spider web not only vibrates in the direction vertical to the spider web plane but also performances slight horizontal vibration along the direction parallel to the web surface. The horizontal vibration of spiral lines is consistent with the longitudinal vibration of nodes, and vibration gradually attenuates from the inside to the outside. By comparative analysis of Figures 7, 8, and 9, one can see that the maximum peak-to-peak value of longitudinal vibration of nodes is about 7 times than that of the maximum peak-to-peak value of horizontal vibration of spiral lines. Nodes within layers 1-5 of spiral lines undergo horizontal vibration, and amplitude of vibration decreases from the inside out; there is no obvious horizontal vibration of nodes on layers 6-11 of the spiral lines. This phenomenon indicates that horizontal vibration of nodes is related to the distance from the center and the longitudinal amplitude. When the node is located in the inner layer and longitudinal amplitude is greater than 5 cm, the horizontal vibration will be produced, and the increase of longitudinal vibration will strengthen the transverse vibration; when the node is on the outer layer and the longitudinal amplitude is less than 5 cm, attenuation of longitudinal vibration leads to failure to produce horizontal vibration. Through comparison and analysis, it is found that the spreading rule of longitudinal and horizontal vibrations is similar to the adjustment mode of node transmission power in the wireless sensor network. The longitudinal vibration is equivalent to normal transmission power of nodes while the horizontal vibration is similar to dynamic adjustment and increase of node transmission power. Under common conditions, the node mainly transmits information across layers or in the same layer through normal transmission power. Only when the flow rate of the cluster head near the base station exceeds a certain threshold, the node will adjust the transmission power to take on more traffic.

**Figure 10:**
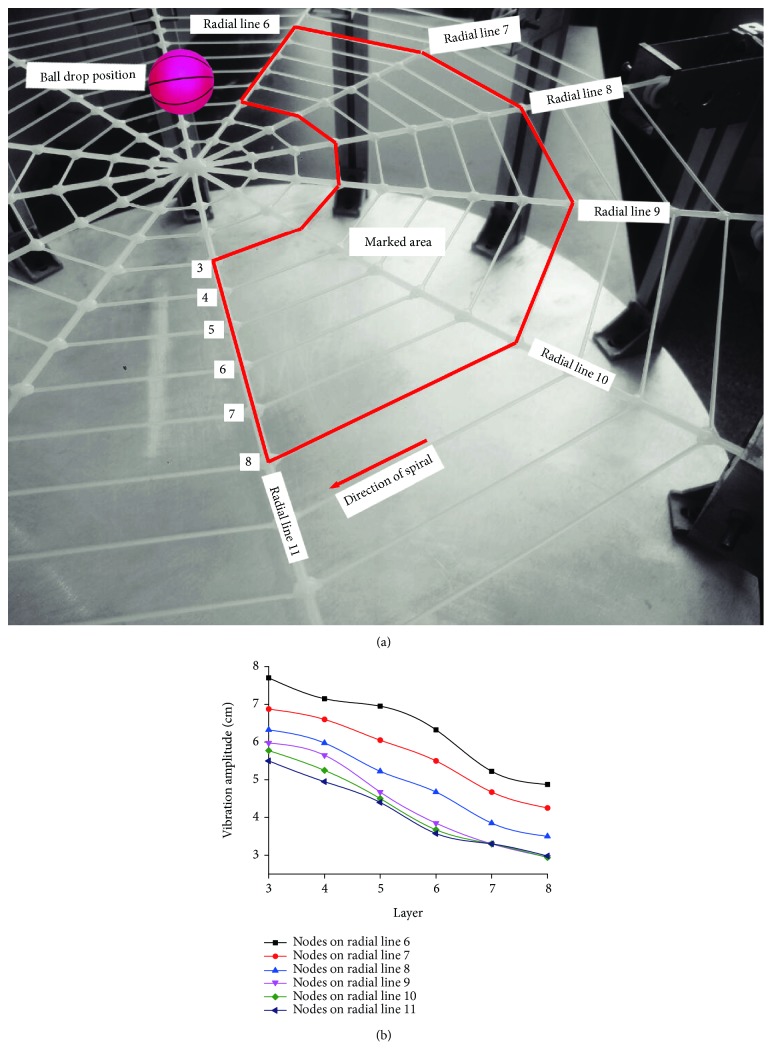
Variation of longitudinal vibration of 36 nodes at intersection of spiral lines on layers 3-8 and radial lines 6-11 in response to central vibration source. (a) Distribution of nodes tracked; (b) Variation curve of vibration peak-to-peak values of nodes tracked.

The longitudinal vibration performance of 36 nodes at intersection of spiral lines on layers 3-8 and radial lines 6-11 in response to central vibration source is shown in Figure 10. It can be seen that the vibration rule of nodes on radial lines of the same layer is roughly the same; peak-to-peak values of vibration decrease in the direction of amplification along the spiral line, and peak-to-peak values of vibration on the same radial line decrease from the inner to outer layer. That is, the farther away from the base station, the more obvious the attenuation trend of vibration. The test result shows that the artificial spider web features the same vibration characteristics among its nodes on the same layer; this phenomenon is similar to the layered wireless sensor network with a center; according to MEBC protocol, nodes communicating with the base station through the same minimum number of hops are located in the same layer, and nodes on the same layer have approximate communication capability and power consumption [29].

### 3.2. Damage Web Test

**Figure 11:**
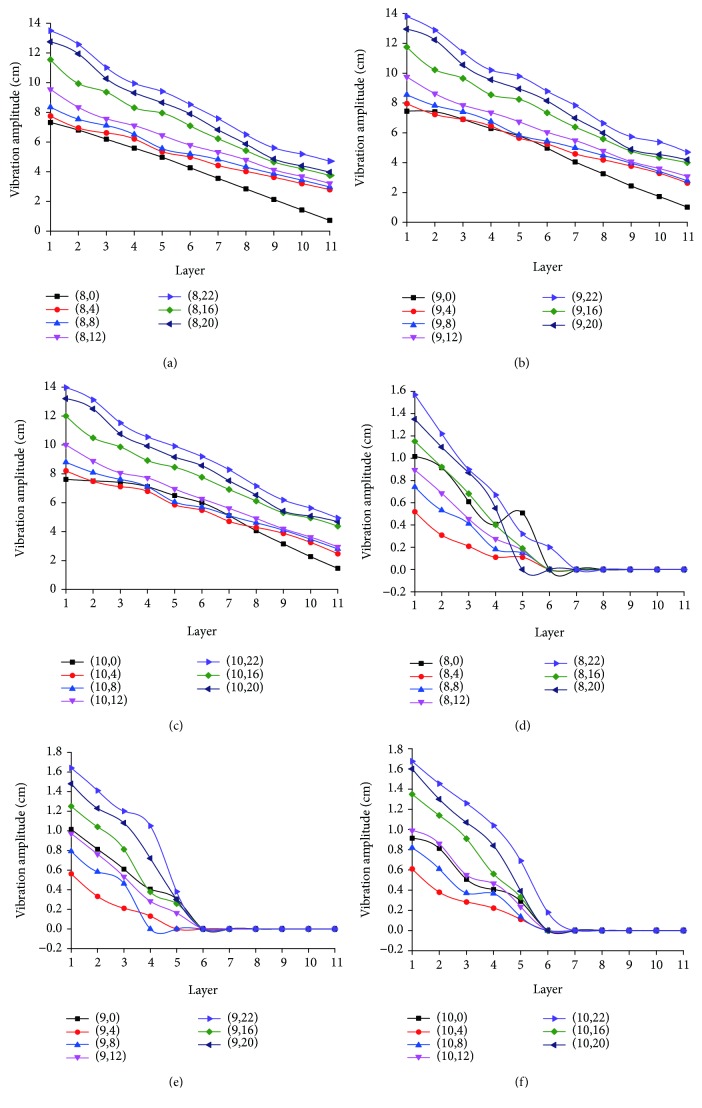
Variation of longitudinal and horizontal vibrations of 33 nodes on radial lines 8-10 when damage occurs. (a) Variation curve of longitudinal vibration peak-to-peak value of nodes on radial line 8; (b) variation curve of longitudinal vibration peak-to-peak value of nodes on radial line 9; (c) variation curve of longitudinal vibration peak-to-peak value of nodes on radial line 10; (d) variation curve of horizontal vibration peak-to-peak value of nodes on radial line 8; (e) variation curve of horizontal vibration peak-to-peak value of nodes on radial line 8; (f) variation curve of horizontal vibration peak-to-peak value of nodes on radial line 8; (8,0), (9,0), and (10,0), respectively, represent variation curves of longitudinal and horizontal vibration peak-to-peak values of radial line 8, radial line 9, and radial line 10 when the web is sound.

**Figure 12:**
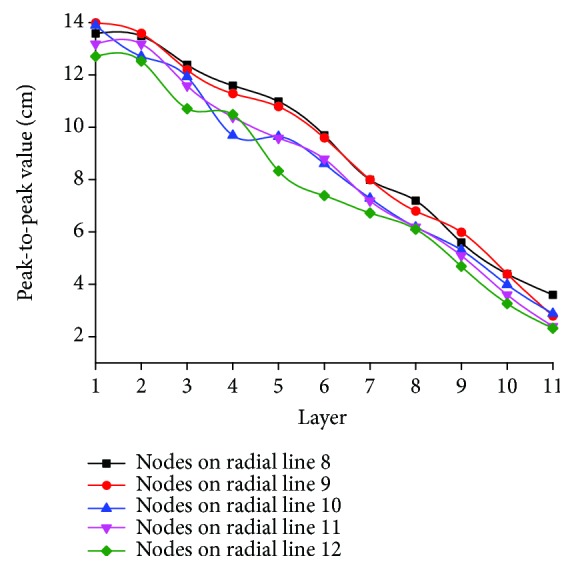
Curve of longitudinal vibration of nodes on radial lines 8, 9, 10, 11, and 12 when damage occurs.

Figure 11 shows variation of peak-to-peak values of longitudinal and horizontal vibrations when nodes at intersection of radial lines 6-7 and spiral lines 1-2, spiral lines 3-4, spiral lines 5-6, spiral lines 7-8, spiral lines 9-10, and spiral line 11 are cut off in turn, based on tracking of longitudinal and horizontal vibrations of 33 nodes on radial lines 8-10. It shows that as the number of cut-off nodes increases, the artificial spider web's vibration intensity increases gradually, and they present a significant positive correlation (*r* ^2^ ≥ 0.954, *p* ≤ 0.01). When radial lines 6-7 are destroyed completely, the peak-to-peak values of longitudinal vibration and horizontal vibration of the same node are increased by nearly 1 time and 0.6 times, respectively, compared with those for a complete network. The results show that before the artificial spider web is damaged, impact of the small ball is borne by all nodes of the whole network. After some nodes are cut off, the remaining nodes bear the impact of the ball, vibration presents an increasing trend, and the inner nodes show stronger increase.  Figure 12 shows the curve of peak-to-peak values of longitudinal vibration of nodes on radial lines 8, 9, 10, 11, and 12 in response to change of the layer number when radial lines 6 and 7 are cut off. It can be seen that from the vicinity of the damage area to the location far away from the damage area, peak-to-peak values of vibration of nodes on the same layer decrease gradually. The above phenomenon indicates that when nodes are damaged seriously, the artificial spider web can adjust independently and limit the influence to vibration transmission of the peripheral area to a certain range for completing effective transmission of the vibration information. The specific adjustment is as follows: when a few nodes are damaged, peak-to-peak value of longitudinal vibration increases, thus effectively sharing the horizontal vibration of spiral lines 1-5 layers and reducing peak-to-peak value of horizontal vibration; when the number of damaged nodes increases, increase of longitudinal vibration cannot further reduce the impact of damage, so more vibration information will spread horizontally, and their peak-to-peak values will increase. The vibration transmission mode of the artificial spider web in response to node damage presents strong guiding significance for research on invulnerability in the wireless sensor network. That is, in a wireless sensor network, when the damage degree of the network is low, by reasonably enhancing information processing capability of a few key nodes (cluster head or cross-layer routing node), influence of network damage can be effectively reduced, but when the network damage degree is high, it is necessary to add more key nodes and common nodes to undertake information transmission in order to effectively eliminate the adverse effects of network damage. At the same time, this test is only a preliminary exploration of invulnerable communication of the wireless sensor network inspired by the artificial spider web. More accurate dynamic theories and quantitative analysis of vibration information transmission, which are transplantable to wireless sensor network, will need further tests and studies.

**Figure 13:**
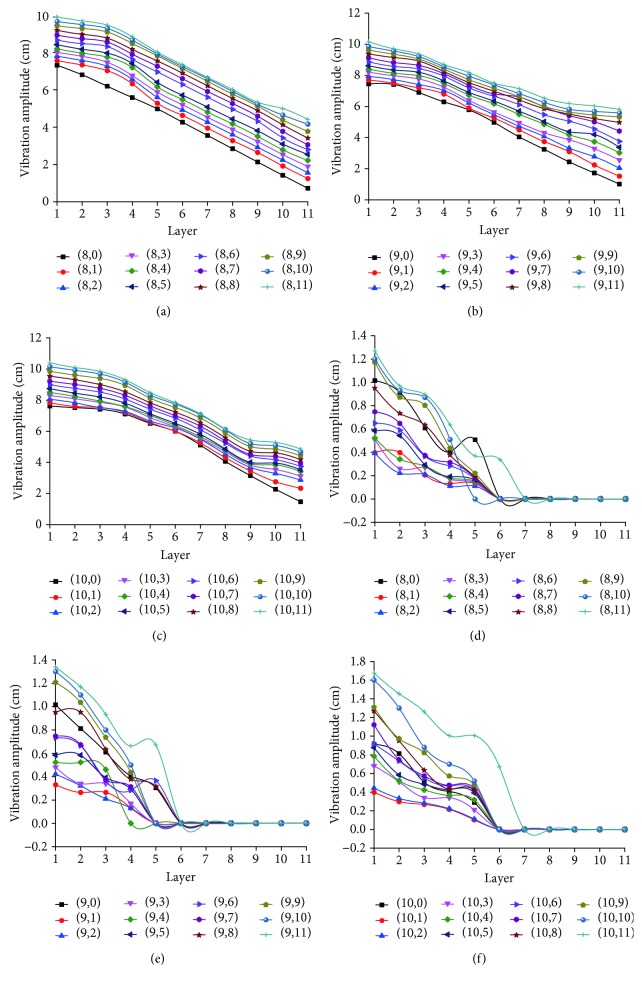
Variation of peak-to-peak values of longitudinal and horizontal vibrations of 33 nodes on radial lines 8-10 when spiral lines are damaged. (a) Variation curve of longitudinal vibration peak-to-peak value of nodes on radial line 8; (b) variation curve of longitudinal vibration peak-to-peak value of nodes on radial line 9; (c) variation curve of longitudinal vibration peak-to-peak value of nodes on radial line 10; (d) variation curve of horizontal vibration peak-to-peak value of nodes on radial line 8; (e) variation curve of horizontal vibration peak-to-peak value of nodes on radial line 9; (f) variation curve of horizontal vibration peak-to-peak value of nodes on radial line 10; (8,0), (9,0), and (10,0), respectively, represent variation curves of longitudinal and horizontal vibration peak-to-peak values of radial line 8, radial line 9, and radial line 10 when the web is sound.

Figure 13 indicates variation of peak-to-peak values of longitudinal and horizontal vibrations of the tracked 33 nodes on radial lines 8-10 when spiral lines 1-11 are cut off in turn. It shows that as the number of cut-off spiral lines increases, artificial spider web's vibration intensity increases gradually, and they present a significant positive correlation (*r* ^2^ ≥ 0.978, *p* ≤ 0.01). When spiral lines are cut off layer by layer, the longitudinal peak-to-peak value of the same node increases by about 0.25 cm with each one layer cut off, accounting for about 3.3% of the amplitude of the whole web. The variation rule of horizontal vibration is similar to that of node cutting off. When the number of cut-off spiral parts reaches 7-8 layers, the increase of radial vibration cannot continue to reduce the impact of damage, so more vibration information will spread horizontally, and its peak-to-peak value of vibration will tend to increase. When the 11 layers of spiral lines are completely cut off, peak-to-peak values of radial vibration and horizontal vibration of the same node are increased by about 0.2 times and 0.3 times, respectively, compared with those of the complete web. It can be known from comparison with damaged nodes that, when spiral lines are damaged, the increase rate and intensity of peak-to-peak values of nodes at the same position are much weaker. This indicates that the proportion of vibration borne by spiral lines is very small, and vibration is mainly transmitted by radial line paths. Spiral lines, as an auxiliary path, bear a small part of the vibration. When nodes and spiral lines are cut off separately, cutting off nodes makes much greater impact on the whole web's vibration than cutting off spiral lines does. Vibration performance of the artificial spider web under the condition of spiral line failure presents wconsiderable reference for study of invulnerability of the wireless sensor network. That is, in a wireless sensor network, communication links between nodes on the same layer can assist information transmission, and their damage will add to the burden of cross-layer communication borne by peripheral nodes; with the increase of the degree of network damage, the role of communication links between nodes on the same layer should be gradually enhanced for reducing the impact of network damage; the communication links between cluster heads and cross-layer routing nodes should receive more attention and protection than the communication links between nodes on the same layer. Effective combination of the two kinds of communication links can ensure network communication capability in case of local damage. In the future, more attention should be paid to distribution of artificial spider web nodes, spiral lines, and radial lines as well as the effect of distribution density on vibration transmission performance. Network topology inspired by the artificial spider web should be optimized under the premise of ensuring relatively strong invulnerability.

**Figure 14:**
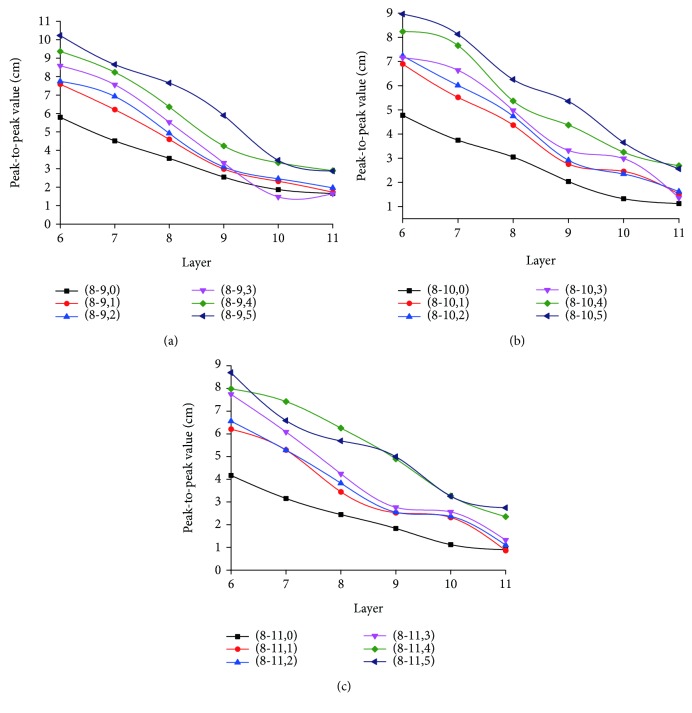
Variation of longitudinal vibration of spiral lines of layers 6-11 between radial lines 8 and 11 when damage occurs. (a) Curve of longitudinal vibration variation of radial lines 8-9; (b) curve of longitudinal vibration variation of radial lines 9-10; (c) curve of longitudinal vibration variation of radial lines 10-11; (8-9,0), (9-10,0), and (10-11,0), respectively, represent variation curves of horizontal vibration peak-to-peak values of radial lines 8-9, radial lines 9-10, and radial lines 10-11 when the web is sound.

When spiral lines of layers 1-5 are cut off layer by layer, the curve of vibration variation of spiral lines 6-11 between radial lines 8 and 11 is shown in Figure 14. It shows that as the spiral lines cut off increases from layer 1 to layer 5, the vibration peak-to-peak values of spiral lines on layers 6, 7, 8, and 9 grow by about 4 cm while that of such lines on layers 10 and 11 grow by about 2 cm. This indicates that along with increase in spiral lines cut off, vibration on the remaining spiral lines will rise; the increase amplitude of the outermost 2 layers is smaller than that of other layers while spiral lines and nodes come with similar mechanism in response to damage.

## 4. Conclusion

The special biological topology of the spider web in nature greatly inspires the research on invulnerability of the wireless sensor network. Based on test results in this paper, we can find that the artificial spider web is amazingly similar to the layered wireless sensor network with a center in many aspects such as the topology structure and information transmission mode. When an artificial spider web sustains serious damage to its nodes and links, the residual structure of the web can still undertake effective vibration information transmission for the whole web. The excellent invulnerability of the artificial spider web presents important reference for the wireless sensor network's topology construction, evaluation of critical nodes or links, layered routing protocol development, and so on. Probing into and summarizing the vibration transmission rule of the artificial spider web may provide brand new method guidance for research of invulnerable communication. The further work should make in-depth analysis of the mapping relationship between vibration transmission capability and structural factors and establish the dynamic theory for vibration information transmission of the spider web. Moreover, the optimized model of the artificial spider web should be abstracted by means of quantitative and qualitative analyses, and the network topology construction and routing method should be built for the wireless sensor network inspired by the spider web.

## Data Availability

The simulation data used to support the findings of this study are included within the paper.

## Supplementary Materials

Supplementary Materials Figure 1: potential application analysis of spider web vibration transmission in the wireless sensor network.
