# Supplementary material for: Exploring Vibration Transmission Rule of an Artificial Spider Web for Potential Application in Invulnerability of Wireless Sensor Network
Source: Appl Bionics Biomech. 2019 May 19;2019:5125034. doi: 10.1155/2019/5125034 (PMC6545770; doi:10.1155/2019/5125034)
Supplement: Supplementary Materials — Figure 1: potential application analysis of spider web vibration transmission in the wireless sensor network. [file 5125034.f1.pdf]

Exploring vibration transmission rule of artificial spider web for potential application in invulnerability of wireless sensor network

A bionic test device based on 3D printing was established to explore the possibility of referring to vibration transmission rule of spider web for improving the invulnerability in wireless sensor network. Two types of experiments were respectively implemented to investigate vibration transmission characteristics of artificial spider web under conditions of integrity and destruction by high-speed photography system. The related test results have huge potential inspiration value for wireless sensor network in terms of routing strategy, network deployment, and topology construction, etc.

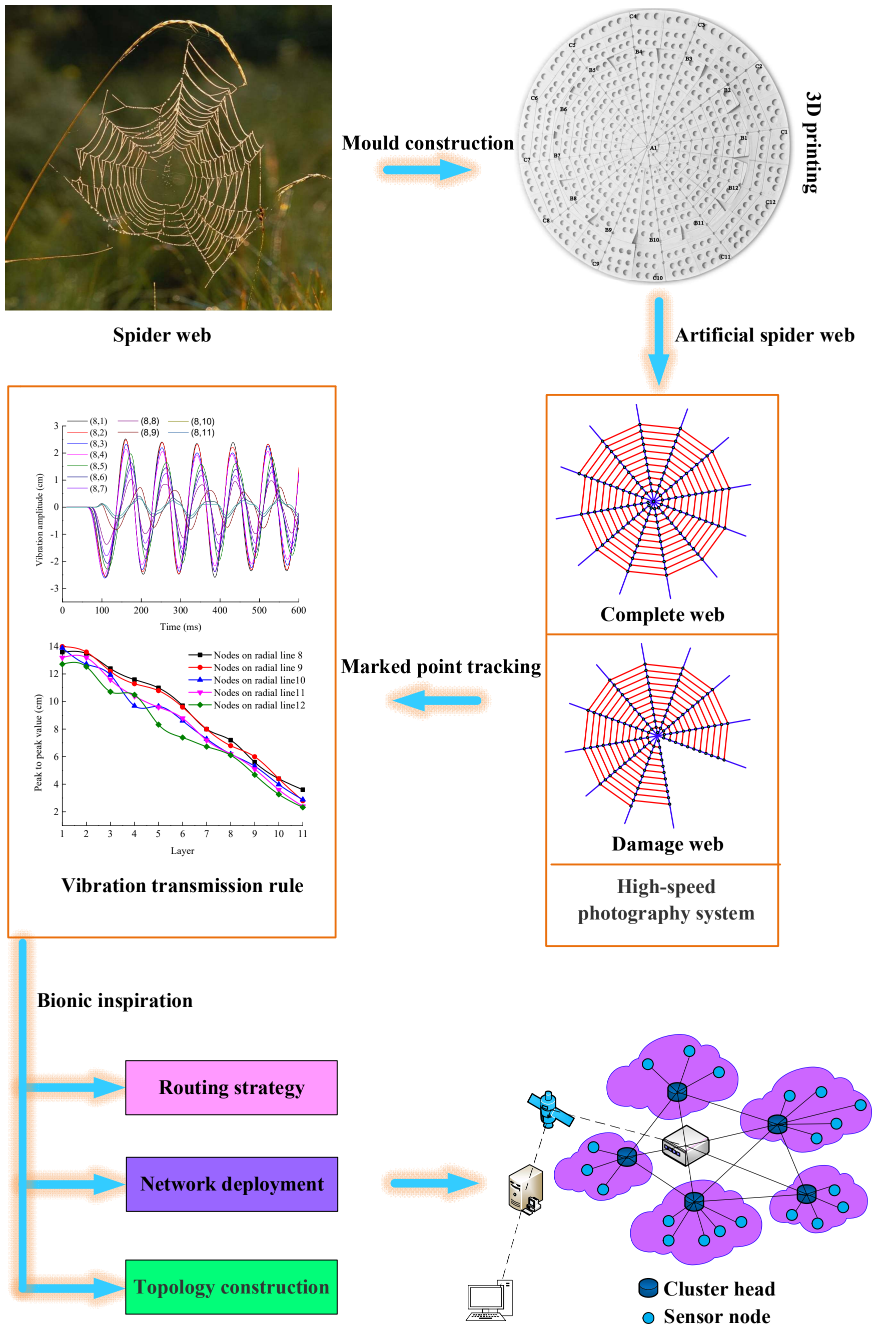

Figure. 1 Potential application analysis of spider-web vibration transmission in wireless sensor network.
